# Cerebrospinal Fluid Biomarkers in Opioid Dependence: Evidence of Neuroimmune Activation and Ion Composition Changes, Without Alteration in Orexin‐A

**DOI:** 10.1111/adb.70053

**Published:** 2025-06-02

**Authors:** Tim Lyckenvik, Malin Woock, Kalle Johansson, Markus Axelsson, Henrik Zetterberg, Kaj Blennow, Eric Hanse, Pontus Wasling

**Affiliations:** ^1^ Department of Physiology Institute of Neuroscience and Physiology, Sahlgrenska Academy at the University of Gothenburg Gothenburg Sweden; ^2^ Department of Neurology Sahlgrenska University Hospital Gothenburg Sweden; ^3^ Department of Clinical Neuroscience Institute of Neuroscience and Physiology, Sahlgrenska Academy at the University of Gothenburg Gothenburg Sweden; ^4^ Department of Psychiatry and Neurochemistry Institute of Neuroscience and Physiology, Sahlgrenska Academy at the University of Gothenburg Mölndal Sweden; ^5^ Clinical Neurochemistry Laboratory Sahlgrenska University Hospital Mölndal Sweden; ^6^ Wisconsin Alzheimer's Disease Research Center, School of Medicine and Public Health University of Wisconsin‐Madison Madison United States; ^7^ Department of Neurodegenerative Disease UCL Institute of Neurology London UK; ^8^ UK Dementia Research Institute at UCL London UK; ^9^ Hong Kong Center for Neurodegenerative Diseases Clear Water Bay Hong Kong China; ^10^ Paris Brain Institute, ICM, Pitié‐Salpêtrière Hospital, Sorbonne University Paris France; ^11^ Neurodegenerative Disorder Research Center, Division of Life Sciences and Medicine, and Department of Neurology Institute on Aging and Brain Disorders, University of Science and Technology of China and First Affiliated Hospital of USTC Hefei China

## Abstract

Opioid abuse is a severe global health challenge, leading to rising morbidity, mortality, and increasing societal costs. The aim of this study was to investigate neuroinflammation, neuronal damage and potential changes in the orexin system or beta‐amyloid metabolism in the cerebrospinal fluid (CSF) of individuals undergoing opioid substitution therapy (OST). This cross‐sectional study investigates CSF biomarkers in individuals undergoing OST, compared to control subjects. Participants receiving OST were recruited from the outpatient clinic at the Department of Psychiatry, Sahlgrenska University Hospital, Gothenburg (Sweden). Each participant provided a complete medical history, including details of drug use over the past 6 months, followed by a lumbar puncture to obtain CSF samples. Molecules associated with neuroinflammation, neuronal and glial damage, beta‐amyloid metabolism and orexinergic function were analysed in the participants' CSF, alongside electrolyte levels. Specifically, we analysed levels of sTREM‐2, YKL‐40, IL‐1β, IL‐6, IL‐8, IL‐10, TNF‐α, AXL, MER, TYRO3, GAS6, NfL, GFAP, total tau (T‐tau), phosphorylated tau (P‐tau), neurogranin, Aβ40, Aβ42, the Aβ42/Aβ40 ratio, orexin‐A, sPDGFR‐β and electrolytes. The study included 15 control subjects and 17 in the opioid substitution group. Patients undergoing opioid substitution therapy exhibited elevated levels of sTREM‐2, Aβ42/Aβ40 ratio and NfL in their CSF. Conversely, concentrations of Na^+^ and Cl^−^ were lower compared to controls. No significant differences were found between groups for other biomarkers, including orexin‐A. However, when normalized to Aβ40 levels, YKL‐40, IL‐8, TYRO3 and P‐Tau were also elevated in individuals with opioid dependence. Elevated biomarkers of neuroimmune activation, neuronal damage and beta‐amyloid metabolism in opioid dependence suggest CNS inflammation as a contributor to its pathophysiology. Reduced electrolyte levels imply disrupted CSF water regulation, possibly linked to impaired glial function. These findings highlight both neural and non‐neural mechanisms in opioid dependence.

## Graphical abstract

Cerebrospinal fluid from individuals with opioid dependence shows elevated neuroinflammatory biomarkers, indicating CNS immune activation.

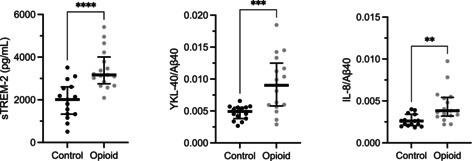

## 1 Introduction

The opioid crisis remains one of the most pressing global public health challenges, with devastating consequences for individuals and society alike. The misuse of opioids, including prescription medications like fentanyl and oxycodone as well as illicit drugs like heroin, has led to a rise in addiction, overdose deaths and associated societal and healthcare costs [1]. Opioid substitution therapy (OST), using medications like buprenorphine and methadone, is a cornerstone of treatment for opioid use disorder (OUD). Although effective at reducing cravings and withdrawal symptomatology, thus preventing relapse and risk behaviour [2], OST does not address the underlying neurobiological changes caused by chronic opioid use.

The mesolimbic dopamine system is well established as the neural circuitry underlying addiction [3], and emerging evidence suggests that non‐neuronal cells like microglia and astrocytes also play a critical but underexplored role in the pathophysiology of opioid dependence through neuroinflammation. Activation of these glial cells triggers the release of pro‐inflammatory cytokines and immune mediators, which can modulate reward pathways and potentially exacerbate the addictive process [4].

Opioids have been shown to induce inflammation both peripherally and centrally. In humans, opioid use has been linked to elevated serum levels of pro‐inflammatory cytokines such as TNF‐α, IL‐1β, IL‐6 and interferon‐gamma (IFNγ) [5], alongside increased release of anti‐inflammatory cytokine release, such as IL‐4, IL‐10 and TGFβ [6]. Within the CNS, opioids predominantly elicit pro‐inflammatory responses. Postmortem studies of individuals with opioid addiction reveal significant upregulation of immune pathways, including activation of microglia, astrocytes and oligodendrocytes [7, 8]. Similarly, animal models demonstrate opioid‐induced glial cell activation and increased expression of pro‐inflammatory markers [4, 9]. Collectively, these findings suggest a role of neuroinflammation in the development of opioid dependence, and potentially in the response to other addictive drugs as well [10, 11].

Despite these findings, human research into neuroinflammation in opioid dependence remains limited, relying heavily on postmortem studies and animal models. This leaves a gap in the knowledge of dynamic immune responses in the living human brain. Biomarkers of neuroinflammation and neuronal damage, reflecting molecular changes within the CNS, can be accessed through cerebrospinal fluid (CSF) sampling, providing an opportunity to address this gap. However, comprehensive investigations of CSF biomarkers in individuals with opioid dependence remain scarce or missing.

This study addresses this gap by analysing CSF biomarkers in individuals undergoing opioid substitution therapy to investigate neuroinflammation, neuronal damage and orexin levels. By leveraging CSF as a window into CNS processes, this work aims to identify novel biomarkers that enhance understanding of the mechanisms underlying opioid addiction and possibly improve the diagnosis of opioid dependence.

## 2 Materials and Methods

### 2.1 Study Design and Participants

This non‐interventional, cross‐sectional, prospective study was conducted at the outpatient substitution clinic within the Department of Psychiatry, Sahlgrenska University Hospital, Gothenburg, Sweden, from November 2019 to July 2020. Eligible participants were adults aged 18–65 years with a confirmed diagnosis of opioid abuse (ICD‐10 code F11.20) according to ICD‐10 criteria. A full diagnostic evaluation was performed by a psychiatrist specialized in drug abuse disorders. All participants (*n* = 17) had been engaged in the medically assisted treatment program for at least 6 months prior to their inclusion in the study. Written and oral informed consent were obtained from all participants.

### 2.2 Medication and Illicit Drug use

Participants were asked about their current use of medications and illicit drugs during the 3 months prior to the study. All participants were undergoing substitution therapy with either buprenorphine or methadone. Information regarding smoking habits and alcohol consumption was not collected.

### 2.3 Saliva and Urine Drug Tests

To evaluate the concurrent use of illicit drugs, medical records were reviewed for urine and saliva drug test results from the 3 months preceding the lumbar puncture, where available. The addictive substances and their metabolites tested in the saliva samples included opioids (*n* = 23), benzodiazepines (*n* = 21), central stimulants (*n* = 19), gabapentin, pregabalin, THC, phencyclidine (PCP), ketamine, mescaline, LSD, psilocin and tianeptine. Urine samples were analysed for buprenorphine, methadone, opiates, oxycodone, tramadol, fentanyl, benzodiazepines, amphetamine, cannabinoids, benzoylecgonine, zolpidem and zopiclone.

### 2.4 Control Subjects

Fifteen healthy controls were recruited from the local community, as previously described [12]. Inclusion criteria for the control group were as follows: age 18–65 years, the ability to provide informed consent, no history of neurological disorders and no substance abuse or use of illicit drugs or medications affecting wakefulness, sleep or cognition. Depression was assessed using the PHQ‐9, and none of the participants scored within the depressive range. Additionally, a review of electronic medical records found no indications of depression or other psychiatric illnesses.

### 2.5 CSF Collection and Analysis

CSF samples were collected via lumbar puncture performed in an upright seated position using a 25G atraumatic needle. Collection occurred during the daytime, between 9 AM and 1 PM. The CSF samples were centrifuged at 2000*g* for 10 min at room temperature to remove cells and debris, then stored in aliquots at −80 °C for future biochemical analyses. All CSF analyses were conducted at the Neurochemistry Laboratory, Sahlgrenska University Hospital, which is accredited by Swedac. Board‐certified technicians performed all analyses.

Soluble triggering receptor expressed on myeloid cells 2 (sTREM‐2) was measured using an enzyme‐linked immunosorbent assay (ELISA) from Uscn Life Science Inc. (Cloud‐Clone Corp., Houston, TX, USA), following the manufacturer's instructions.

Chitinase‐3‐like protein 1 (CHI3L1) (YKL‐40) was quantified using the Human Chitinase 3‐like protein 1 (CHI3L1) Quantikine ELISA Kit (R&D Systems, Minneapolis, Minnesota).

Proinflammatory cytokines (IL‐1β, IL‐6, IL‐8, IL‐10, TNF‐α, IFN‐γ) were analysed using the Meso Scale Discovery 4‐plex Proinflammatory Panel II (Meso Scale Discovery, Rockville, Maryland).

Tyrosine‐protein kinase receptor 3 (TYRO3), tyrosine kinase receptor Axl (AXL), tyrosine kinase Mer (MER) and growth arrest‐specific protein 6 (GAS6) were analysed using the Olink Explore 3072 platform from Olink Proteomics.

Neurofilament light chain (NfL), glial fibrillary acidic protein (GFAP) and neurogranin were measured using validated in‐house ELISA methods [13, 14, 15].

Total tau (T‐tau) and phosphorylated tau (P‐tau) were measured using Lumipulse technology (Fujirebio, Ghent, Belgium).

Aβ‐related biomarkers (Aβ40, Aβ42) were measured using electrochemiluminescence assays (Meso Scale Discovery, Rockville, Maryland, USA).

Soluble platelet‐derived growth factor receptor‐β (sPDGFR‐β) was analysed using a sandwich ELISA (Invitrogen Cat. no. EHPDGFRB, Thermo Fisher Scientific, Loughborough, United Kingdom).

Orexin‐A levels in CSF were measured using an in‐house radioimmunoassay (RIA), with a normal reference range defined as > 400 pg/mL [16].

CSF sodium, potassium and chloride concentrations were measured using ion‐selective electrodes integrated into the cobas c 501 instrument (Roche Diagnostics). Calcium and magnesium concentrations were measured colorimetrically using the o‐cresolphthalein and chlorophosphonazo III methods, respectively. CSF and serum albumin concentrations in control subjects were measured by immunonephelometry on a Beckman Image Immunochemistry system (Beckman Coulter, Brea, California, United States), and the albumin quotient (Qalb) was calculated as CSF albumin (mg/L) divided by serum albumin (g/L).

### 2.6 Ethics

This study was approved by the Regional Board of Medical Ethics at the University of Gothenburg (approval number: #2019‐03061). All participants provided written informed consent before inclusion in the study.

### 2.7 Statistical Analysis

The data were analysed using GraphPad Prism Software (version 10.2.0 for Mac, GraphPad Software, San Diego, California, United States). Data are reported as median and interquartile range (IQR) unless otherwise noted. Group differences in demographic and clinical data were assessed using the Mann–Whitney test due to the skewed distribution observed in many distributions through the Shapiro–Wilk normality test. Fisher's exact test was used to compare dichotomous variables, such as sex, between groups and Spearman's rank correlation was used in correlation analyses. All comparisons were two‐tailed, and statistical significance was set at *p* < 0.05. To account for multiple comparisons, a Bonferroni correction was applied by adjusting the significance threshold. The conventional α‐level of 0.05 was divided by the number of statistical tests performed (N), resulting in an adjusted significance threshold of α/N. This correction was used to minimize the risk of type I errors while maintaining statistical rigour.

## 3 Results

### 3.1 Demography

There were significant differences in the gender and age distribution between the opioid dependence group and the control group. The opioid dependence group had **a** higher proportion of males. In the opioid dependence group, 13 out of 17 participants were male, while in the control group, 4 out of 15 participants were male, *p* = 0.0118) and they were older (opioid dependence: median 38 years, IQR 33.0–48.0; control: median 25 years, IQR 23.0–29.0, *p* < 0.0001). None of the control group participants reported using opioids, benzodiazepines, central stimulants or other medications.

### 3.2 Characteristics of Participants With Opioid Dependence

Demographic details, illicit drug use, prescribed medication and results from saliva and urine samples of subjects on opioid substitution therapy are presented in Table 1. Of the 17 subjects with opioid dependence, 16 subjects were medicated with buprenorphine, a partial agonist of the μ‐opioid receptor, and an antagonist to κ‐ and δ‐opioid receptors. One was medicated with methadone, a μ‐opioid receptor agonist. Thirteen subjects reported using illicit drugs or substances not prescribed by a physician during the last 3 months before lumbar puncture. Five subjects used more than one illicit substance. Four subjects had smoked or injected heroin, in addition to their substitution therapy.

**TABLE 1:** Self‐reported opioid dependence, illicit drug use, drug test results and prescribed medication among participants.

ID	Age (years)	Sex	Reason for substitution	Substitution therapy	Ongoing illicit substances	Drug test	Prescribed medication
1	29	F	Heroin	Buprenorphine	No	Opioids	Melatonin
2	31	M	Heroin	Buprenorphine	Alprazolam	BZD, P/G	Escitalopram, bupropion, mirtazapine, amitriptyline, formoterol, budesonide, omeprazole
3	32	M	Heroin	Buprenorphine	No	Opioids, THC	Quetiapine, melatonin
4	32	F	Heroin	Buprenorphine	Alprazolam	Opioids, BZD, STIM, THC, P/G	Pregabalin
5	34	F	Heroin	Buprenorphine	Heroin, amphetamine injected, alprazolam	Opioids, BZD, STIM	
6	34	M	Heroin	Methadone	Heroin smoked, lisdexamfetamine, alprazolam	Opioids, BZD, STIM, P/G	
7	35	M	Heroin	Buprenorphine	No	Opioids, P/G	Gabapentin, quetiapine
8	38	M	Heroin	Buprenorphine	Heroin smoked/injected, alprazolam, alcohol	Opioids, BZD, THC	Sertraline, olanzapine, mirtazapine, acamprosate
9	38	M	Heroin	Buprenorphine	Heroin smoked, amphetamine, lisdexamfetamine, alprazolam	Opioids, BZD, STIM, THC	
10	39	M	Heroin	Buprenorphine	Alprazolam	Opioids, BZD, STIM, P/G	
11	43	M	Heroin	Buprenorphine	Bedrocan®, Sativex®, ( *Cannabis sativa* )	Opioids, BZD, THC	Mirtazapine, zopiclone, melatonin, duloxetine, valproic acid
12	46	M	Heroin	Buprenorphine	Alprazolam	Opioids, BZD, THC	
13	48	M	Heroin	Buprenorphine	Alprazolam	Opioids, BZD, P/G	Gabapentine, sertraline
14	48	F	Morphine	Buprenorphine	No	Opioids	
15	49	M	Heroin	Buprenorphine	Alprazolam	Opioids, BZD, P/G	Zopiclone
16	57	M	Heroin	Buprenorphine	Alprazolam	Opioids, BZD, STIM	Pregabalin
17	64	M	Heroin	Buprenorphine	BZD, alcohol	Opioids, BZD	

Abbreviations: BZD, benzodiazepine; P/G, pregabaline/gabapentine; STIM, central stimulants.

The most frequently used substances were benzodiazepines, reported by 12 out of 17 subjects, with the primary benzodiazepine being alprazolam. Three subjects reported using both heroin and central stimulants together with benzodiazepines or alcohol. One subject mentioned concurrent use of *Cannabis sativa* .

Given the potential underreporting of illicit substance use, urine and saliva drug tests taken during the 3 months preceding the lumbar puncture were reviewed. The drug test results indicated: opioids, positive in 16 out of 17 subjects; benzodiazepines, positive in 13 out of 17 subjects; central stimulants, positive in 5 out of 17 subjects; THC, positive in 6 out of 17 subjects; gabapentin/pregabalin, positive in 7 out of 17 subjects. Twelve out of 17 subjects tested positive for substances they had not self‐reported, including central stimulants, THC, gabapentin/pregabalin and zopiclone.

### 3.3 Biomarkers

CSF samples were collected from 17 subjects with opioid dependence and 15 control participants. The CSF cell count for neutrophils and lymphocytes was less than 4 × 10^6^/L in all subjects. Monocyte counts were also below 3 × 10^6^/L except in two subjects: #2 and #14 (6 × 10^6^/L and 4 × 10^6^/L, respectively). No pleocytosis was observed in the control group. Due to the absence of blood samples in most cases, selective oligoclonal bands in the CNS could not be analysed.

**FIGURE 1:**
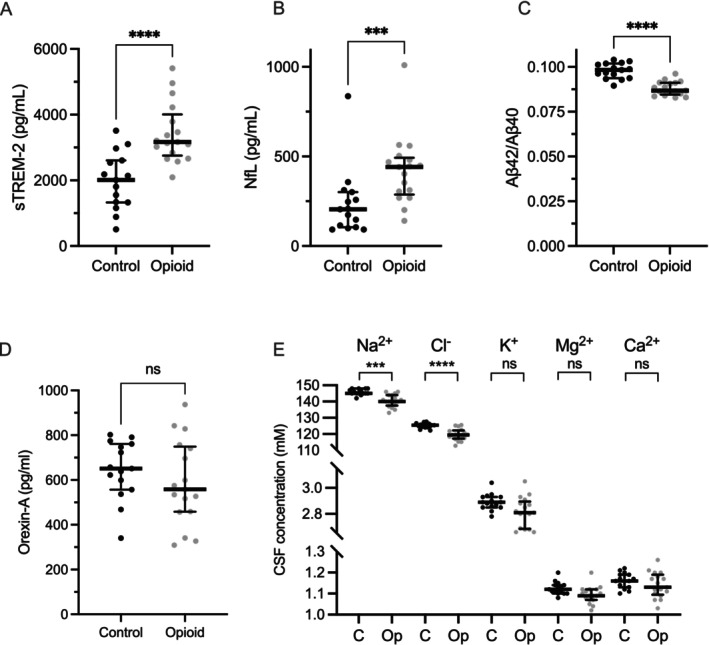
Alterations in cerebrospinal fluid (CSF) biomarkers in individuals with opioid dependence compared to controls. Abbreviations: sTREM‐2, soluble triggering receptor expressed on myeloid cells 2; NfL, neurofilament light chain; Aβ40, amyloid β (1–40); Aβ42, amyloid β (1–42). Data are presented as median and interquartile range. Statistical differences were analysed using the Mann–Whitney test adjusted using the Bonferroni correction for multiple comparisons. Significance levels**:** ****p < 0.0001; ***p < 0.001.

The selected CSF biomarkers were analysed to assess neuroinflammation and potential neuronal damage in the subjects with opioid dependence. Table 2 provides a summary of the analyses, while Figure 1 highlights biomarkers with statistically significant differences between the groups. A Bonferroni correction was applied by adjusting the significance threshold, dividing 0.05 by the number of tests (23), resulting in an adjusted threshold of 0.00217.

**TABLE 2:** Cerebrospinal fluid levels of biomarkers and electrolytes in controls and individuals with opioid dependence.

	Control (*n* = 15)	Opioid (*n* = 17)	Statistical analysis	
	Median (IQR, range)	Median (IQR, range)	Not normalized	Normalized to Aβ40
sTREM‐2 (pg/mL)	2014 (1325–2606, 3005)	3167 (2752–4006, 3319)	**< 0.0001 (**29, 0.77)	**< 0.0001 (**22.5, 0.82)
YKL‐40 (ng/mL)	63.2 (38.4–81.6, 64.8)	73.6 (66.0–109.5, 175.3)	0.0163	**0.0006 (**40, 0.68)
IL‐1β (pg/mL)	Not detectable	Not detectable	NA	NA
IL‐6 (pg/mL)	0.67 (0.60–1.02, 2.38)	0.77 (0.67–1.29, 1.14)	0.345	0.2613
IL‐8 (pg/mL)	29.9 (26.0–37.8, 31.8)	42.3 (33.9–51.3, 39.0)	0.0112	**0.0016 (**46.5, 0.64)
IL‐10 (pg/mL)	Not detectable	Not detectable	NA	NA
TNF‐α (pg/mL)	Not detectable	Not detectable	NA	NA
AXL (pg/mL)	11 580 (8062–12 661, 9764)	10 233 (9577–11 649, 7561)	0.766	0.076
MER (pg/mL)	456.3 (335.7–563.4, 654.6)	437.1 (368.2–484.9, 263.0)	0.508	0.103
TYRO3 (pg/mL)	2078 (908.3–2234, 3418)	2220 (1945–2749, 2116)	0.165	**< 0.0001 (**25.5, 0.8)
GAS6 (pg/mL)	7347 (5319–8592, 5259)	7781 (7012–8947, 4561); *n* = 16	0.151	0.0055
NfL (pg/mL)	205 (105–301, 744)	441 (287–493, 869)	**0.0008 (**42.5, 0.67)	**0.0003 (**37, 0.71)
GFAP (pg/mL)	378 (255–554, 483)	429 (320–634, 865)	0.282	0.036
T‐tau (pg/mL)	289 (210–842, 1850)	268 (224–320, 1849)	0.648	0.350
P‐tau (pg/mL)	29.1 (18.7–40.3, 33.0)	34.0 (29.1–39.2, 28.1)	0.317	**< 0.0001 (**14, 0.89)
Neurogranin (pg/mL)	194 (119–254, 280)	194 (128–214, 214)	0.478	0.545
Aβ40 (pg/mL)	12 262 (8050–15 315, 12 958)	11 537 (7792–12 631, 10 177)	0.245	NA
Aβ42 (pg/mL)	1218 (775–1568, 1376)	997 (681–1109, 978)	0.0486	NA
Aβ42/Aβ40	0.0983 (0.094–0.102, 0.015)	0.087 (0.085–0.091, 0.013)	**< 0.0001 (**11, 0.91)	NA
Orexin‐A (pg/mL)	651 (557–761, 462)	558 (458–749, 628)	0.249	0.689
sPDGFR‐β	282 (187–311, 374)	302 (276–321, 150)	0.205	0.0028
Na^+^ (mmol/L)	145.0 (145.0–148.0, 6.0)	141.0 (138.5–144.5, 13.0)	**0.0004 (**25.5, 0.8)	0.350
K^+^ (mmol/L)	2.89 (2.85–2.93, 0.26)	2.81 (2.68–2.89, 0.39)	0.0289	0.313
Cl^−^ (mmol/L)	125.5 (124.2–126.3, 5.4)	119.5 (117.2–122.2, 12.8)	**< 0.0001 (**22, 0.83)	0.350
Ca^2+^ (mmol/L)	1.16 (1.13–1.19, 0.12)	1.13 (1.09–1.19, 0.23)	0.256	0.261
Mg^2+^ (mmol/L)	1.12 (1.10–1.14, 0.12)	1.10 (1.08–1.12, 0.16)	0.034	0.365

Abbreviations: IQR, interquartile range; sTREM‐2, soluble triggering receptor expressed on myeloid cells 2; YKL‐40, chitinase‐3‐like protein 1 (CHI3L1); IL, interleukin; TNF‐α, tumour necrosis factor α; IFN‐γ, interferon γ; TYRO3, tyrosine‐protein kinase receptor 3; AXL, tyrosine kinase receptor Axl; MER, tyrosine kinase Mer; GAS6, growth arrest‐specific protein 6; NfL, neurofilament light chain; GFAP, glial fibrillary acidic protein; T‐tau, total tau; P‐tau, phosphorylated tau; Aβ40, amyloid β (1–40); Aβ42, amyloid β (1–42); sPDGFR‐β, soluble platelet‐derived growth factor receptor β. Data are presented as median (IQR, range). Statistical comparisons were performed using the Mann–Whitney *U* test, with *p* values adjusted using the Bonferroni correction for multiple comparisons. Corrected *p* values are reported, with *U* values and effect sizes (rank‐biserial correlation, *r*) provided in parentheses after each *p* value. Significant *p* values are shown in bold**.**

Microglial activation marker sTREM2, the astrocytic activation markers YKL‐40 and GFAP, cytokines (IL‐1β, IL‐6, IL‐8, IL‐10, TNF‐α and IFN‐γ) and components of the TAM receptor signalling system (AXL, MER**,** TYRO3 and GAS6) were measured to investigate potential group differences in neuroinflammation. sTREM2 was significantly elevated in the subjects with opioid dependence (*p* < 0.0001), while no significant differences in YKL‐40 or GFAP levels were detected (*p* = 0.0163 and *p* = 0.282, respectively). There were also no differences in the levels of IL‐6 or IL‐8. IL‐1β, IL‐10, TNF‐α and IFN‐γ were either undetectable or, in the case of IFN‐γ, detected in only a few samples above the detection limit. No significant differences in levels of any of the soluble receptor tyrosine kinases (RTKs), AXL, MER, and TYRO3, which regulate CNS immune responses [17], were detected between the groups. Similarly, levels of their ligand, GAS6 (growth arrest‐specific protein 6), were comparable between the groups.

Next, we examined biomarkers for neuronal damage, where NfL levels were significantly higher in the subjects with opioid dependence (*p* = 0.0008). However, no significant differences were observed between the two groups in the concentrations of T‐tau, P‐Tau or the synaptic function marker neurogranin.

### 3.4 Amyloid‐Beta and Orexin‐A Analysis

While neither Aβ40 or Aβ42 levels were significantly different, there was a significantly reduced Aβ42/Aβ40 ratio (*p* < 0.0001). However, the ratio did not reach the clinical threshold for Alzheimer's disease pathology (< 0.72) in either group. No significant differences were observed Orexin‐A levels.

### 3.5 Blood–Brain Barrier Function and Electrolyte Composition

We used soluble platelet‐derived growth factor receptor‐β (sPDGFR‐β) as a surrogate marker for blood–brain barrier **(**BBB) function [18], but could not detect significant differences between the groups. Due to the absence of blood samples from the subjects with opioid dependence, we were unable to measure serum protein levels and thus could not calculate the CSF/serum albumin ratio to assess BBB function. In the control group, sPDGFR‐β levels correlated significantly with the CSF/serum albumin ratio (Spearman *r* = 0.59, *p* = 0.022), supporting the utility of this marker. We found that concentrations of the measured electrolytes were approximately 3% lower in the opioid dependence group and both Na^+^ and Cl^−^ were significantly different (*p* = 0.0004 and *p* < 0.0001, respectively), while the difference in K^+^, Mg^2+^ and Ca^2+^ levels were not statistically significant.

### 3.6 Altered Fluid Homeostasis in Opioid Dependence

The lower levels of ions in the CSF of the subjects with opioid dependence suggests that their CSF may be diluted. To explore how the biomarkers were related to each other, as well as to age and sampling timing, we conducted a correlation analysis for each group (see Figure 3A,C). In the control group, there was a pattern of positive correlations across nearly all measured proteins, peptides, and cytokines. IL‐6 deviated from this pattern by showing inverse correlations with other organic molecules. The electrolytes also consistently displayed positive correlations with each other, separately from the organic molecules. In the subjects with opioid dependence, the typical correlations among neuroinflammatory markers were weaker, while the relationships largely persisted among RTKs, amyloid β markers and orexin (Figure 3C).

### 3.7 Uncovering Group Differences Through Normalization of Biomarkers to Amyloid‐β 40

Correlation analysis revealed that CSF hydration accounts for much of the variability in biomarker levels among controls but not in the opioid group. To address group differences masked by CSF hydration variability, biomarker concentrations were normalized to CSF water content using Aβ40 as the reference protein, an analysis that was recently proposed [19]. Aβ40 strongly correlates with median brain‐derived protein levels. This suggests that Aβ40 serves not only as a normalization factor for the Aβ42/Aβ40 ratio but also as a broader indicator of overall CSF protein levels, reflecting individual‐specific CSF dynamics.

**FIGURE 2:**
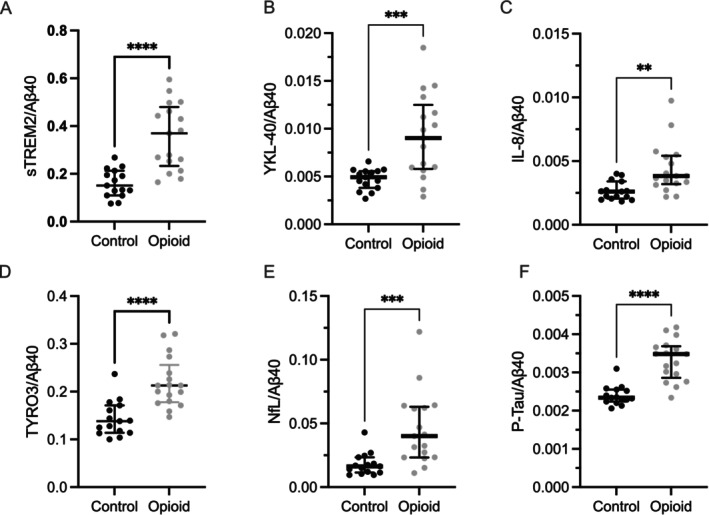
Significant alterations in cerebrospinal fluid (CSF) biomarkers in individuals with opioid dependence compared to controls, normalized to Aβ40. Abbreviations: sTREM‐2, soluble triggering receptor expressed on myeloid cells 2; Aβ40, amyloid β (1–40); YKL‐40, chitinase‐3‐like protein 1 (CHI3L1); IL, interleukin; NfL, neurofilament light chain; P‐tau, phosphorylated tau; TYRO3, tyrosine‐protein kinase receptor 3. Data are presented as median and interquartile range. Statistical differences were analysed using the Mann–Whitney test adjusted using the Bonferroni correction for multiple comparisons. Significance levels**:** ****p < 0.0001; ***p < 0.001; **p < 0.01.

Normalization uncovered additional significant group differences, with YKL‐40, IL‐8, TYRO3 and P‐tau levels elevated in the opioid group (Figure 2, Table 2), further indicating neuroimmune activation in opioid dependence. While normalization reduced *p* values for most biomarkers, significance for ion concentrations was lost, suggesting a partially compensated dilution effect in the opioid group.

All biomarkers that showed significant differences between groups (i.e. sTREM, NfL, Aβ42/40 ratio, sTREM/Aβ40, YKL‐40/Aβ40, IL‐8/Aβ40, TYRO3/Aβ40, NfL/Aβ40 and P‐Tau/Aβ40) were assessed for correlation with daily buprenorphine dose using Spearman correlation analysis. No significant associations were observed (*p* values 0.77, 0.19, 0.74, 0.99, 0.97, 0.14, 0.78, 0.37 and 0.58).

**FIGURE 3:**
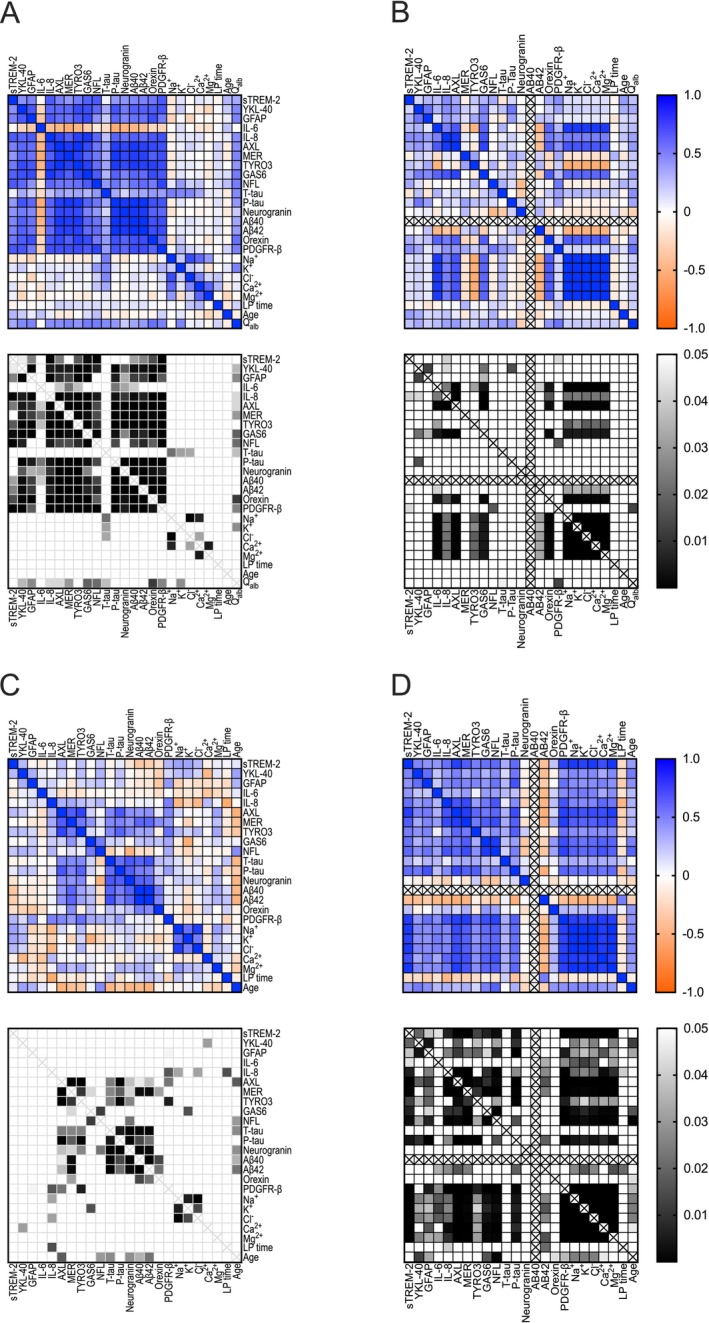
Correlations between CSF biomarkers in opioid‐dependent and control groups, with and without normalization to Aβ40. (A) Spearman correlation (*r*) and *p* values for control group. (B) Spearman correlation (*r*) and *p* values for control group, normalized to Aβ40. (C) Spearman correlation (*r*) and *p* values for opioid substitution group. (D) Spearman correlation (*r*) and *p* values for opioid substitution group, normalized to Aβ40. In controls, most proteins, peptides and cytokines showed positive correlations, except IL‐6, which was inversely correlated with other markers. Electrolytes clustered separately with strong internal correlations. In the opioid group, correlations among neuroinflammatory markers were weaker, while associations among RTKs, amyloid β markers, and orexin persisted. Abbreviations: sTREM‐2, soluble triggering receptor expressed on myeloid cells 2; YKL‐40, chitinase‐3‐like protein 1 (CHI3L1); IL, interleukin; TYRO3, tyrosine‐protein kinase receptor 3; AXL, tyrosine kinase receptor Axl; MER, tyrosine kinase Mer; GAS6, growth arrest‐specific protein 6; NfL, neurofilament light chain; GFAP, glial fibrillary acidic protein; T‐tau, total tau; P‐tau, phosphorylated tau; Aβ40, amyloid β (1–40); Aβ42, amyloid β (1–42); sPDGFR‐β, soluble platelet‐derived growth factor receptor beta; Qalb, albumin quotient; S‐Alb, serum albumin concentration; CSF‐Alb, CSF albumin concentration; LP Time, time of day for lumbar puncture. Data representation: Spearman correlation coefficients (*r*) range from −1 to 1; *p* values range from < 0.01 to 0.05.

In controls, normalization reduced correlations between organic molecules, addressing CSF water content's confounding effects (Figure 3B). However, normalized ion concentrations were not only strongly correlated with each other, but also with orexin, IL‐6, IL‐8, AXL and GAS6 while showing inverse correlations with normalized TYRO3, MER and Aβ42, indicating interconnected physiological processes between neuroimmune activation, BBB permeability regulation, amyloid metabolism and ion homeostasis.

In the opioid group (Figure 3D), distinct correlations linked neuroinflammatory markers, PDGFR‐β, ion concentrations, P‐tau and NfL, suggesting heightened neuroimmune activation, BBB permeability and neuronal injury. Neurogranin and Aβ42 inversely correlated with these markers, possibly reflecting altered amyloid metabolism and synaptic dynamics in this group.

## 4 Discussion

This cross‐sectional study examined CSF profiles in individuals with opioid dependence undergoing substitution therapy compared to healthy controls, identifying biomarkers of neuroinflammation (sTREM‐2), neuronal injury (NfL) and altered amyloid metabolism. Electrolyte levels (Na^+^ and Cl^−^) were significantly lower, with no differences in orexin‐A. After normalization to Aβ40, additional elevations in markers of neuroinflammation (YKL‐40, IL‐8 and TYRO3), and neuronal injury (P‐Tau) emerged. These findings indicate chronic neuroimmune activation, neuronal injury, and disrupted amyloid β dynamics as neurobiological consequences of chronic opioid use.

### 4.1 Evidence for Neuroinflammation in Opioid Dependence

We observed elevated CSF levels of sTREM2, and, after normalization to water content, also increases in YKL‐40, IL‐8 and TYRO3 in the opioid dependence group. These findings align with both postmortem human studies [7, 8] and preclinical rodent models [9], which show increased glial activation and release of pro‐inflammatory cytokines in opioid addiction.

sTREM2 is predominantly expressed in microglia where it regulates key microglial functions like proliferation, migration, survival and phagocytosis. Elevated CSF sTREM2 levels have been associated with neurological diseases such as Alzheimer's disease and multiple sclerosis [20, 21]. Similarly, YKL‐40 serve as markers for astrocyte activation. YKL‐40 also modulates microglial activity and has been linked to neuroinflammation in conditions such as Alzheimer's disease, ALS, HIV and multiple sclerosis [22, 23, 24]. Similarly, previous studies have suggested that opioids can induce GFAP expression in the brain [9, 25], but the difference in GFAP did not reach statistical significance in this study.

IL‐8, primarily released by microglia and endothelial cells, plays a central role in mediating neuroinflammation and promoting phagocytosis [26]. Meanwhile, the TAM receptor TYRO3 and its ligand GAS6 function to regulate the inflammatory response. Activation of TAM receptors, including TYRO3, is known to suppress the inflammatory cascade. The elevated levels of TYRO3 observed in the opioid dependence group may therefore reflect an adaptive response aimed at controlling ongoing inflammation.

Taken together, our findings suggest that opioid dependence is associated with neuroimmune activation, evidenced by elevated markers of glial activation (sTREM2 and YKL‐40), increased IL‐8 and the activation of regulatory pathways involving TYRO3.

### 4.2 Theoretical Framework for Opioid Dependence Development Through Microglial Activation

Opioids exhibit a strong binding affinity to toll‐like receptor 4 (TLR4), a pattern recognition receptor predominantly expressed on microglia, with lesser expression on astrocytes [27]. TLR4 is typically activated by microbial components, such as lipopolysaccharides (LPS), but morphine can also directly activate the TLR4‐MD2 complex. This activation triggers a cascade of neuroinflammatory signalling, including the release of pro‐inflammatory cytokines, which may disrupt neurotransmission and contribute to the development of morphine tolerance, a key mechanism underpinning opioid dependence [28].

In addition to TLR4 activation, microglia can be stimulated via their μ‐opioid receptor, which opioids like morphine and buprenorphine target directly, leading to the release of pro‐inflammatory cytokines which may affect neurotransmission and synaptic plasticity, potentially further contributing to the development of opioid dependence [29]. Most subjects in the study were on substitution therapy with buprenorphine, a partial agonist at the μ‐opioid receptor. This partial activation may perpetuate mild neuroinflammatory responses, although the extent of these theoretical effects remains unknown, especially compared to full agonists like heroin or morphine.

Microglia are involved in clearing extracellular amyloid‐β deposits in Alzheimer's disease [30], and their activation can induce hyperphosphorylation, aggregation and spread of tau protein [31, 32]. Therefore, microglial activation may explain the lower levels of Aβ42/Aβ40 ratio and relatively higher levels of P‐tau seen in our study.

### 4.3 Possible Neuronal Damage in Opioid Dependence

Neuroinflammation has been linked to neuronal degeneration in diseases like Alzheimer's, HIV, multiple sclerosis, Parkinson's and amyotrophic lateral sclerosis [33, 34]. Neurofilament light chain protein (NfL) is an intermediate filament used to assess axonal damage across neurological disorders [35]. This study found significantly higher NfL levels in CSF compared to controls, suggesting nerve cell damage related to opioid dependence. However, previous studies in mice indicated a downregulation of NfL following chronic opioid exposure [36, 37] and NfL is known to increase with age and to being higher in men [38], complicating interpretations in this study where age and sex distribution is skewed between groups. Moreover, only P‐tau, not T‐tau nor neurogranin, show significant differences between groups as to further indicate neuronal damage.

### 4.4 CSF Dilution in Opioid Dependence and Its Impact on Biomarker Levels

Electrolyte levels were about 3% lower in the subjects with opioid dependence, although the K^+^, Mg^2+^ and Ca^2+^ concentrations were not statistically significant, indicating a redistribution of water to the extracellular space. Disruptions in osmotic homeostasis can lower CSF Na^+^ and Cl^−^ through BBB dysfunction, impaired ion transporters (Na^+^‐K^+^ ATPase, Na^+^‐Cl^−^ cotransporters), astrocytic uptake and metabolic acidosis. Glial dysfunction, including cytokine‐induced transporter suppression and disrupted AQP4 function, may further contribute to ion imbalance. Hypothetically, microglial activation may trigger regulatory volume decrease mechanisms, e.g. via release of ATP [39] or reactive oxidative species [40], inducing release of organic molecules along with water from cells, contributing to electrolyte dilution. After normalizing for CSF water content, no measured ion concentration significantly differed between groups, supporting dilution, rather than selective ion concentration regulation, as the mechanism for lower electrolyte concentrations.

The non‐significant trend in relatively higher sPDGFR‐β among subjects with opioid dependence could possibly indicate a parallel disruption in BBB integrity, consistent with previous studies linking neuroinflammation, specifically astrocytic activation, to BBB disruption [41, 42]. However, ion concentrations appear to be largely independent of BBB permeability, indicating that BBB disruption is not the primary cause of CSF dilution in the opioid group.

### 4.5 No Difference in Orexin Concentration Between Groups

No significant differences in orexin‐A levels between the opioid and control groups were found neither before nor after normalization to Aβ40. Previous postmortem studies on opioid users showed a 50% increase in orexin‐producing neurons in the hypothalamus [43]. We therefore hypothesized that subjects suffering from opioid dependence would exhibit higher CSF orexin levels, which was not substantiated in this study. Given orexin‐A's role in motivation and reward, an increase might be expected. However, buprenorphine treatment may not elevate orexin‐expressing neurons, and chronic opioid use may not necessarily lead to increased orexin‐A levels. The absence of an observed increase could also be due to a small sample size and limited statistical power.

### 4.6 Limitations

This study has several limitations. The main limitation of this study is the underpowered sample size. Larger, more adequately powered studies are needed to confirm the results. Age and sex were also not matched between groups, potentially explaining some proportion of the biomarker differences. Moreover, many participants used multiple CNS‐active drugs, and group differences in smoking and alcohol habits were not assessed, making it difficult to isolate opioid effects. Another limitation is that the self‐reported drug use may result in selection bias. Furthermore, the cross‐sectional approach prevents establishing causal links between substance abuse, neuroinflammation, or ion composition changes and the sample size may be too small to detect all significant differences and is not sufficient to determine diagnostic cut‐offs for biomarkers. We cannot determine with certainty whether the observed increases in biomarker levels are primarily driven by the underlying OUD or by the effects of substitution therapy. However, it is plausible that agonistic activation of opioid receptors contributes to the observed biomarker alterations. Further studies with controlled designs are needed to clarify these associations. Four subjects were prescribed medications primarily used to treat mood and anxiety disorders, psychotic and bipolar disorders, alcohol dependence, and chronic pain or migraine. As these disorders are known to be associated with neuroinflammation, this should be taken into account when interpreting the results. It is also challenging to control for potential covariates that may influence biomarkers of neuroinflammation and neuronal damage, such as comorbid conditions, medication use, lifestyle factors or undetected substance use, which may confound the observed associations. Despite these limitations, this study highlights the possible role of innate immune activation in opioid dependence and underscores the value of CSF biomarkers for investigating neuroinflammatory pathways.

## 5 Conclusions

In summary, our findings from the first study of CSF biomarkers in human subjects with opioid addiction suggest that opioid dependence is associated with the activation of microglia and astrocytes in the CNS, leading to the release of the pro‐inflammatory cytokine IL‐8 and RTK‐activation. Additionally, we observed signs of altered Aβ metabolism and tau hyperphosphorylation, possible axonal damage, and dysregulation of water homeostasis in the CSF of individuals undergoing opioid substitution therapy. The activation of glial cells may contribute to the development of addiction by releasing pro‐inflammatory cytokines that may affect neural transmission. Therefore, targeting neuroinflammation in patients with substance dependence disorders may offer a promising treatment approach.

## Author Contributions

**Tim Lyckenvik:** writing – original draft, data analysis. **Malin Woock:** writing – original draft, data collection, data analysis. **Kalle Johansson:** data collection, writing – review and editing. **Markus Axelsson:** data collection, writing – review and editing. **Henrik Zetterberg:** data analysis, writing – review and editing, funding acquisition. **Kaj Blennow:** data analysis, writing – review and editing, funding acquisition. **Eric Hanse:** writing – review and editing, funding acquisition. **Pontus Wasling:** conceptualization, methodology, original draft, data analysis, data collection, supervision, funding acquisition.

## Conflicts of Interest

HZ has served at scientific advisory boards and/or as a consultant for Abbvie, Acumen, Alector, Alzinova, ALZPath, Amylyx, Annexon, Apellis, Artery Therapeutics, AZTherapies, Cognito Therapeutics, CogRx, Denali, Eisai, LabCorp, Merry Life, Nervgen, Novo Nordisk, Optoceutics, Passage Bio, Pinteon Therapeutics, Prothena, Red Abbey Labs, reMYND, Roche, Samumed, Siemens Healthineers, Triplet Therapeutics, and Wave, has given lectures sponsored by Alzecure, BioArctic, Biogen, Cellectricon, Fujirebio, Lilly, Novo Nordisk, Roche and WebMD, and is a co‐founder of Brain Biomarker Solutions in Gothenburg AB (BBS), which is a part of the GU Ventures Incubator Program (outside submitted work). KB has served as a consultant and at advisory boards for Abbvie, AC Immune, ALZPath, AriBio, BioArctic, Biogen, Eisai, Lilly, Moleac Pte. Ltd, Neurimmune, Novartis, Ono Pharma, Prothena, Roche Diagnostics and Siemens Healthineers; has served at data monitoring committees for Julius Clinical and Novartis; has given lectures, produced educational materials and participated in educational programs for AC Immune, Biogen, Celdara Medical, Eisai and Roche Diagnostics; and is a co‐founder of Brain Biomarker Solutions in Gothenburg AB (BBS), which is a part of the GU Ventures Incubator Program, outside the work presented in this paper. MA has received compensation for lectures and/or advisory boards from Biogen, Genzyme, Merck and Novartis. The other authors have nothing to disclose.

## Data Availability

Data available on request from the authors.
